# Inducing forgetting of unwanted memories through subliminal reactivation

**DOI:** 10.1038/s41467-022-34091-1

**Published:** 2022-10-30

**Authors:** Zijian Zhu, Michael C. Anderson, Yingying Wang

**Affiliations:** 1 School of Psychology, Shaanxi Normal University, Xi’an, 710062 Shaanxi China; 2 MRC Cognition and Brain Sciences Unit, University of Cambridge, Cambridge, CB27EF UK; 3 Department of Psychology and Behavioral Sciences, Zhejiang University, Hangzhou, 310028 Zhejiang China

**Keywords:** Human behaviour, Forgetting

## Abstract

Processes that might facilitate the forgetting of unwanted experiences typically require the actual or imagined re-exposure to reminders of the event, which is aversive and carries risks to people. But it is unclear whether awareness of aversive content is necessary for effective voluntary forgetting. Disrupting hippocampal function through retrieval suppression induces an amnesic shadow that impairs the encoding and stabilization of unrelated memories that are activated near in time to people’s effort to suppress retrieval. Building on this mechanism, here we successfully disrupt retention of unpleasant memories by subliminally reactivating them within this amnesic shadow. Critically, whereas unconscious forgetting occurs on these affective memories, the amnesic shadow itself is induced by conscious suppression of unrelated and benign neutral memories, avoiding conscious re-exposure of unwelcome content. Combining the amnesic shadow with subliminal reactivation may offer a new approach to voluntary forgetting that bypasses the unpleasantness in conscious exposure to unwanted memories.

## Abstract (web summary)

Classical forgetting methods typically re-expose people to reminders of their unwanted memories. Here, the authors disrupt unpleasant memories by subliminally reactivating them as participants suppress retrieval of unrelated neutral memories, avoiding the need for conscious exposure.

## Introduction

Recurrent intrusive memories and ruminations are key symptoms in a range of psychiatric conditions, including post-traumatic stress disorder (PTSD), acute stress disorder, and obsessive-compulsive disorder^1^. Treatments on these symptoms often emphasize gradual re-exposure to the major stressors. For instance, a widely used therapy for PTSD, exposure therapy, involves gradually confronting cues related to the traumatic event; and a second, the eye movement desensitization and reprocessing (EMDR) treatment approach requires that patients hold a mental image of the traumatic event in mind while visually tracking a bilateral stimulus^2^. Actual or imaginal re-exposure, although effective in reducing symptoms, can be aversive to patients. Reponses to aversive content can lead participants to prematurely terminate therapy and also involve additional risks to patients^3,4^. Here we ask whether it is possible to reduce the intrusiveness of an unwanted memory while avoiding any requirement for people to consciously reexperience it.

To address the foregoing problem, we propose that an unwanted memory may be forgotten by subliminally reactivating it during a time window when hippocampal processing is actively inhibited by voluntary retrieval suppression. Such unconscious forgetting can be accomplished by modifying procedures for studying retrieval suppression. This surprising possibility follows from what is known about the neural mechanisms underlying retrieval suppression^5–7^ and unconscious memory processing^8,9^. Research on retrieval suppression has found that intentionally suppressing (i.e., stopping) memory retrieval given a reminder to a memory downregulates hippocampal activity; recent studies suggest that, in doing so, retrieval suppression could globally disrupt hippocampal functions such as the encoding, retrieval and stabilization of memories^5^. Disrupting hippocampal processes mimics organic amnesia, triggering both retrograde and anterograde memory deficits. This effect, known as the amnesic shadow^6,10^, occurs in the temporal surround of each retrieval suppression attempt (extending at least 5–10 s before and after suppression), creating a window during which either recently encoded or older, reactivated “innocent bystander” memories can be disrupted. To be affected by the amnesic shadow, however, a memory’s retention must rely on ongoing hippocampal processing that gets prevented by suppression^10,11^. Critically, evidence suggests that hippocampal traces may be reactivated without awareness. Indeed, the hippocampus mediates rapid associative memory retrieval without requiring consciousness and can be activated even by subliminally presented cues^8,12,13^. Together, these findings imply a striking possibility: it should be possible to forget a hippocampally dependent memory by subliminally exposing reminders to it during the amnesic shadow induced by retrieval suppression on independent memories.

**Fig. 1:**
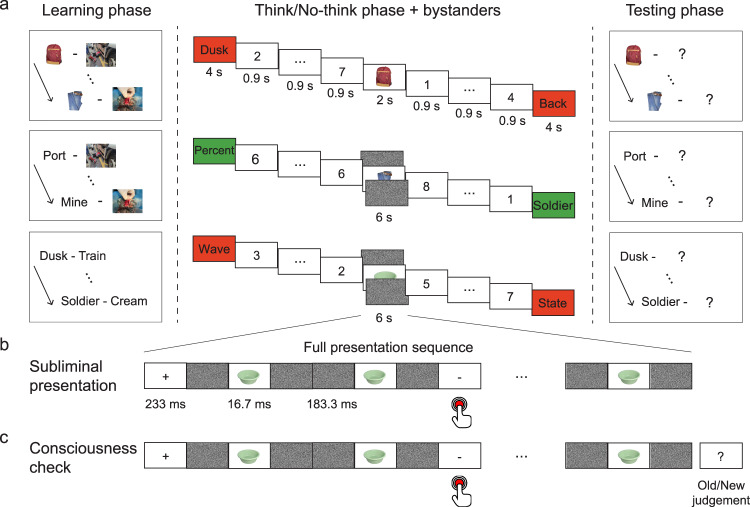
Experimental procedure. **a** Participants learned three series of cue-target associations with the first two series constituting the Bystander pairs (in series 1, the scenes were linked to object cues; in series 2, the same scenes were linked to word cues) and the third series, the TNT pairs. Participants then performed trials involving retrieval (Think, shown in green color) or retrieval suppression (No-think, shown in red color) on TNT pairs. Inserted between every two Think or No-think trials were repeated presentations of a “Bystander” object cue, from one of the Bystander pairs. Think Bystanders and half of the No-think Bystanders were reactivated subliminally with a masking procedure (middle section, **a**); Bystanders between the other half of the No-think trials were presented supraliminally for 2 s (middle section, top). Participants were asked to recognize the supraliminally presented Bystander objects without reporting and performed a memory-irrelevant task (see **b**) during subliminal presentations. Even/odd buffer judgements on numbers were performed before and after Bystander cues to match the immediate task context surrounding Bystander items across Think and No-think trials. At the end, the Bystander cues and then the TNT cues appeared and participants reported the corresponding targets (panel **a**, right side). **b** Procedure for the subliminal reactivation. The whole series, which lasted 6 s, involved a fixed procedure containing six repetitions of the following events: a 233 ms fixation cross, four 183.3 ms white noise masks, and two 16.7 ms cue pictures. Occasionally, the fixation cross would change to a horizontal/vertical line and participants detected the change by key pressing. **c** Consciousness check at the end of the experiment. The consciousness check used the same subliminal presentation procedure as in the Think/No-think phase. The only difference was that after each trial, participants instead judged whether they could identify the masked cue object, and whether the object was old or new in the experiment.

To test this hypothesis, we measured whether retrieval suppression affected the accessibility of memories that were subliminally cued during the amnesic shadow. To induce suppression, we adapted the Think/No-think (TNT) paradigm^14^. In our TNT task, people performed trials in which they received a reminder of a previously studied verbal memory item and were cued either to retrieve the associated word (Think trials) or to suppress its retrieval (No-think trials). Repeated No-think practice has been found to induce forgetting on the suppressed memories, a phenomenon known as suppression-induced forgetting^14^. Importantly, in prior work, these same suppression trials also are known to induce forgetting on entirely unrelated memories (hereinafter called Bystander memories) encoded or reactivated close in time to No-think trials^5^. Therefore, retrieval suppression does not merely disrupt the particular suppressed memories, but may reflect a broadly targeted suppression of activity within the hippocampal region^6^. Here, we inserted subliminal reminders (simple visual objects) to previously encoded Bystander memories (scenes) in between two No-think or two Think trials (see Fig. 1) to maximize the chances that hippocampal processes would be affected during the reminder. Because the previous and subsequent No-think trials should disrupt hippocampal processes, Bystander reminders presented between these trials should fall within the amnesic shadow. Bystander reminders appeared subliminally with a sandwich masking procedure^8,15^. Unbeknownst to participants, target scenes associated with the Bystander objects presented between Think or between No-think trials would be tested after the TNT task, along with target scenes whose reminders were not presented during the TNT task serving as a baseline control (see Fig. 1).

Here, we show that subliminally exposing reminders of unpleasant Bystander scenes during the amnesic shadow impairs participants’ later ability to recall those scenes on the delayed recall test, despite participants having no awareness of the content of the masked reminders during the preceding Think/No-think task. Critically, forgetting on the delayed test not only arises when we test the scene with the same reminder cue used for subliminal reactivation, but also with an independent cue never subliminally re-exposed during the Think/No-think task, suggesting that bystander forgetting reflects the generalized disruption of the reactivated scene memory itself.

## Results

### Forgetting without awareness verified by an offline awareness test

In Experiment 1, we tested whether the amnesic shadow could disrupt a Bystander memory that was subliminally reactivated by reminder cues. We applied a masking procedure to all the Think and to half of the No-think Bystanders so that participants could not consciously perceive the content of Bystander cues (Fig. 1b). For comparison, we presented half of the No-think Bystanders supraliminally as in our previous study^10^. Because we only used the Bystander object cues to reactivate the Bystander memory (Fig. 1b), we refer to the object cues as the Trained cues. In contrast, we refer to Bystander word cues, which were free of direct memory reactivations, as the Independent cues. We performed an offline consciousness check at the end of the experiment to verify that participants could not identify masked Bystander cues (Fig. 1c).

**Fig. 2:**
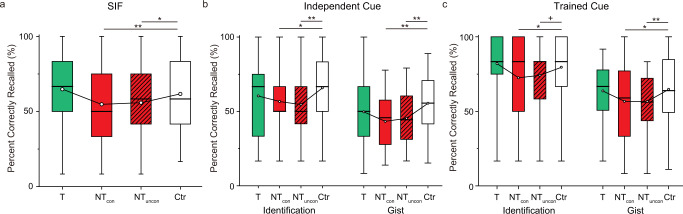
Suppression- and shadow-induced forgetting from Experiment 1. **a** Percentage of targets recalled for the Think/No-think (TNT) pairs (*n* = 40). Voluntary suppression consistently disrupted recall performance, showing suppression-induced forgetting in the conscious (t(39)= −3.11, *p* = 0.003, Cohen’s d = 0.49) and unconscious (t(39)= −2.52, *p* = 0.016, Cohen’s d = 0.40) No-think conditions (T, Think; NT_con_, No-think conscious; NT_uncon_, No-think unconscious; Ctr, Control). **b** Percentage of Bystander images identified and percentage of gist information recollected under independent-cue retrieval of Bystander pairs. Both conscious (Identification, t(39)= −2.68, *p* = 0.011: Cohen’s d = 0.42; Gist, t(39)= −3.67, *p* < .001, Cohen’s d = 0.58) and unconscious (Identification, t(39)= −3.21, *p* = 0.003, Cohen’s d = 0.5; Gist: t(39)= −3.10, *p* = 0.004, Cohen’s d = 0.49) memory reactivation within the amnesic shadow induced by No-think trials impaired later recall of Bystander Scenes. **c** Percentage of Bystander images identified and percentage of gist information recollected under trained-cue retrieval of Bystander pairs. Both conscious (Identification: t(39)= −2.43, *p* = .020, Cohen’s d = 0.38; Gist: t(39)= −2.46, *p* = .019, Cohen’s d = 0.39) and unconscious (Identification: t(39)= −1.96, *p* = .057, Cohen’s d = 0.31; Gist: t(39)= −2.87, *p* = .007, Cohen’s d = 0.45) memory reactivation within the amnesic shadow induced by No-think trials impaired later recall of Bystander Scenes. The boxes in box plots show the inter-quartile range (IQR) and the median. Whiskers in box plots represent the minimum and maximum in the dataset. White dots represent the means for each condition. The asterisks represent significant differences (+*p* < 0.06; *p < 0.05, **p < 0.01, Two-tailed *t* test). Source data are provided in a Source Data file.

We first tested the standard suppression-induced forgetting (SIF) effect on the TNT pairs themselves, verifying our manipulation. Recall accuracy varied significantly across our four conditions (i.e., Think, No-think conscious, No-think unconscious, and Control) (Fig. 2a, F(3,117) = 8.85, *p* < 0.001, η_p_^2^ = 0.19, see also Supplementary Table 1 for details). As expected, retrieval suppression induced significant memory impairment on No-think items when compared with the Control condition (No-think conscious vs. Control: t(39)= −3.11, *p* = 0.003, Cohen’s d = 0.49; No-think unconscious vs. Control: t(39)= −2.52, *p* = .016, Cohen’s d = 0.40), showing that our retrieval suppression manipulation succeeded. Whereas retrieval during Think trials numerically increased final test performance for Think items, the improvement was not significant (Think vs. Control: t(39)= 1.50, *p* = .141, Cohen’s d = 0.24).

Of key interest is whether subliminally exposing Bystander cues within the window of the amnesic shadow would lead to an amnesic shadow effect on the later recall test. Before testing this, it was critical to exclude participants who might have identified or recognized the Bystander cues, despite our subliminal procedure. For this, we turned to the consciousness-checking phase at the end of the experiment during which participants were overtly directed to identify the masked object cues and then make old/new judgement on them. On this task, if a participant could recognize 66.7% or greater (a one-tailed 5% cut-off of 66.7%) of masked objects they were considered likely to have perceived Bystander objects in the earlier TNT phase and so were excluded and replaced. Two participants were excluded and replaced with this procedure. On this basis, the overall recognition accuracy in the Consciousness Checking task in the final sample was 50.30% (Table 1), which was not different from the chance level of 50%. The d’ of the old/new recognition was −1.43, indicating that participants could not recognize the masked stimuli. We note that this exclusion standard is conservative in that participants in the consciousness-checking phase were directed to intentionally identify and recognize masked objects as their main task, which they were not asked to do in the earlier TNT phase.

**Table 1:** Percentage of items that participants claimed to see (%)

	Overall	Think	No-think	New
**Experiment 1**	21.56	25.42	20.50	19.17
**Experiment 2**	12.62	18.06	14.93	4.86

Having eliminated participants who could have identified the subliminal Bystander items, we then tested whether unconscious Bystander reactivation led to an amnesic shadow. The scene targets contained complex affective content allowing us to measure not only whether the scene was recalled (Identification), but also the level of detail that was accessible (Gist). We conducted a 2 (cue type: trained cue vs. independent cue) × 4 (suppression status: Think, No-think conscious, No-think unconscious, and Control) repeated measures ANOVA on both the Identification and Gist accuracy of the Bystander targets (Fig. 2b, c, see also Supplementary Table 2 for details) separately. For Identification accuracy, the main effects of suppression status (F(3,117) = 6.16, *p* < 0.001, η_p_^2^ = 0.14) and cue type (F(1,39) = 28.36, *p* < 0.001, η_p_^2^ = 0.42) were both significant. The two factors did not interact (F(3,117) = 1.27, *p* = 0.29, η_p_^2^ = 0.03), showing similar amnesic shadow effects, irrespective of whether we tested people with the cue used to reactivate the Bystander scene or not. Considering that the trained and independent cues received different treatments – with the trained cues exposed during the shadow period and the independent cues not – we tested the effect of each cue type separately. We found a significant main effect of suppression status for both the independent- (F(3,117) = 3.87, *p* = 0.011, η_p_^2^ = 0.09) and trained-cue tests (F(3,117) = 4.10, *p* = 0.008, η_p_^2^ = 0.10). Replicating our previous findings, supraliminally reactivating Bystanders within the amnesic shadow led to memory impairment, relative to Control items (the cues for which were not re-exposed) and this memory disruption arose irrespective of whether participants were tested with the re-exposed (trained cue) or the independent cue (independent-cue retrieval: t(39)= −2.68, *p* = .011, Cohen’s d = 0.42; trained-cue retrieval: t(39)= −2.43, *p* = 0.020, Cohen’s d = 0.38). Critically, this amnesic shadow effect also occurred for subliminally reactivated Bystanders (independent-cue retrieval: t(39)= −3.21, *p* = 0.003, Cohen’s d = 0.51; trained-cue retrieval: t(39)= −1.96, *p* = 0.057, Cohen’s d = 0.31). Interestingly, shadow-related forgetting did not differ reliably in magnitude between the conscious and unconscious conditions (*p*s > 0.50). In contrast, re-activating Bystander scenes between two Think trials did not reliably affect memory performance for the Bystanders, relative to memory for Control items (independent-cue retrieval: t(39)= 1.45, *p* = 0.156, Cohen’s d = 0.23; trained-cue retrieval: t(39)= 0.78, *p* = 0.438, Cohen’s d = 0.12). Thus, participants’ later ability to recall an aversive scene significantly declined when that scene had been cued subliminally in the window between two retrieval-suppression trials, consistent with an unconscious amnesic shadow effect.

For Gist accuracy, the same 2 by 4 repeated measures ANOVA revealed significant main effects for suppression status (F(3,117) = 6.71, *p* < 0.001, η_p_^2^ = 0.15) and cue type (F(1,39) = 15.88, *p* < .001, η_p_^2^ = 0.29). When inspecting the Trained and Independent Cue performance separately, both showed a significant main effect of suppression status (independent-cue retrieval: F(3117) = 5.02, *p* = .003, η_p_^2^ = 0.11; trained-cue retrieval: F(3,117) = 4.26, *p* = 0.007, η_p_^2^ = 0.10). In line with the findings in the Identification measure, both supraliminal memory reactivation (independent-cue retrieval: t(39)= −3.67, *p* < 0.001, Cohen’s d = 0.58; trained-cue retrieval: t(39)= −2.46, *p* = 0.019, Cohen’s d = 0.39) and subliminal reactivation (independent-cue retrieval: t(39)= −3.10, *p* = 0.004, Cohen’s d = 0.49; trained-cue retrieval: t(39)= −2.87, *p* = 0.007, Cohen’s d = 0.45) within the amnesic shadow impaired later recall of the reactivated Bystander scenes, compared to recall for Control items that were not reactivated. These effects occurred regardless of whether scenes were recalled from trained- or independent-cues, illustrating that memory for the scene was disrupted, independent of the cue used. Therefore, subliminally presenting an unwanted emotional memory within the amnesic shadow window impaired people’s ability to recall key details related to the scene’s meaning.

Objective consciousness analysis based on post hoc selection suffers from problems such as regression to the mean, so we checked participants’ subjective consciousness. Although objective recognition accuracy was at the chance level on our Consciousness Check, participants still reported identifying masked items occasionally. Overall, 23.96% of the Bystander cues (Tables 1, 1.53 out of the 6 Think Bystanders and 1.35 out of the 6 No-think unconscious Bystanders) were reported to be visible. However, when asked whether the putatively identified items were previously studied, the old/new recognition accuracy for the “consciously” perceived items that were previously studied (i.e., old items) was only 76% (Table 2). This suggests that participants may have been adopting a liberal strategy in reporting consciousness of the masked item, sometimes reporting visibility when there was none. If so, this liberal strategy would imply that unreported items are likely not perceived. Building on this possibility, we tested whether the amnesic shadow effects would remain even when we excluded all Bystander items reported visible during the Consciousness Check phase.

**Table 2:** Old/New recognition accuracies for items participants claimed to see or to not see (%)

	Reported seen			Reported unseen		
	Think old	No-think old	New	Think old	No-think old	New
Experiment 1	75.93	76.67	21.59	28.63	30.38	68.84
Experiment 2	95.30	87.00	69.10	30.00	26.10	73.00

On average, 1.35 out of the 6 unconscious No-think Bystanders were excluded. For the remaining items that could not be identified during the Consciousness Check, identification was still disrupted by the amnesic shadow under the independent-cue retrieval (No-think unconscious vs. Control: t(39)= −2.53, *p* = .015, Cohen’s d = 0.40), though not under the trained-cue retrieval (No-think unconscious vs. Control: t(39)= −1.49, *p* = 0.145, Cohen’s d = 0.23). We observed an amnesic shadow effect on our Gist measure on both independent- (No-think unconscious vs. Control: t(39)= −2.45, *p* = 0.019, Cohen’s d = 0.39) and trained-cue (No-think unconscious vs. Control: t(39)= −2.49, *p* = 0.017, Cohen’s d = 0.39) tests. These findings provide evidence for an unconscious amnesic shadow: even when we restricted analyses to only those items that people couldn’t consciously identify when they were intentionally trying, retrieval suppression disrupted Bystander memories that were reactivated close in time.

**Fig. 3:**
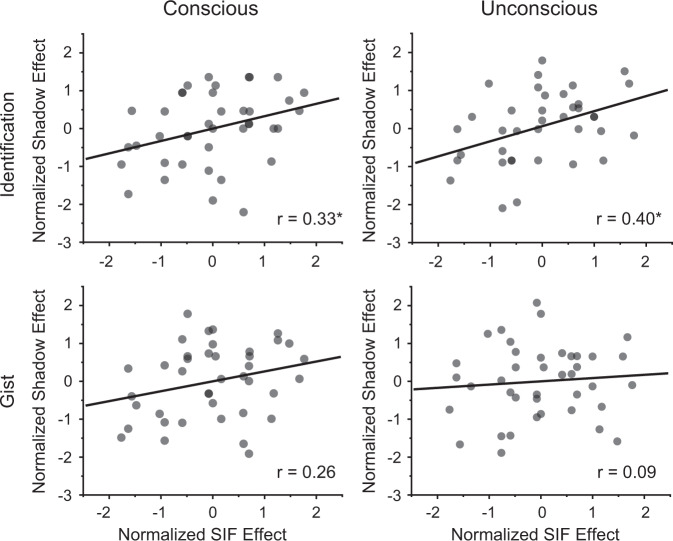
Correlation between the suppression- and shadow-induced forgetting effects. (Top) The degree of suppression-induced forgetting on TNT pairs predicted the shadow effect in the conscious (left, r-skipped = 0.33, [0.07, 0.57] bootstrapped 95% CI) and unconscious (right, r-skipped = 0.40, [0.15, 0.60] bootstrapped 95% CI) condition on the Identification measure. (Bottom) The correlation was not detected for the Gist measure. The asterisk (*) represents statistical significance at *p* < 0.05. Source data are provided in a Source Data file.

Based on the hypothesis that retrieval suppression triggers both hippocampal down-regulation and SIF, the magnitude of SIF may be related to the amnesic shadow^6^. We tested whether SIF on the TNT pairs predicted the amnesic shadow effect on the Bystander scenes. We performed a Pearson correlation between the SIF effect (i.e., Control – No-think) and its shadow effect on Bystanders (i.e., Control – No-think) on both the trained- and independent-cue tests using a robust statistical approach as described by Pernet et al.^16^. Both effects were *z*-normalized within each item counterbalancing condition to account for item-effects, as in prior work^17–19^. Consistent with our hypothesis, on the Identification measure, SIF correlated with the overall shadow effect (averaged over the Trained and Independent Cues) in the No-think conscious condition (Fig. 3 left, r-skipped = 0.33, [0.07, 0.57] bootstrapped 95% CI). A significant correlation also was observed for the No-think unconscious condition after eliminating potential conscious items based on the Consciousness Check performance (Fig. 3 right, r-skipped = 0.40, [0.15, 0.60] bootstrapped 95% CI). However, the same correlation was not detected in the Gist measure for either the conscious (r-skipped = 0.26, [−0.04, 0.57] bootstrapped 95% CI) or the unconscious condition (r-skipped = 0.09, [−0.24, 0.38] bootstrapped 95% CI). Overall, successful SIF was linked to both the conscious and unconscious shadow effect, despite the word pairs used in the TNT and Bystander scenes being entirely unrelated to one another, consistent with the possibility that retrieval suppression had set in motion processes that disrupted scene retention.

### Forgetting without awareness verified by an online awareness test

The findings of Experiment 1 suggest that unconsciously reactivating a memory during the amnesic shadow induces significant forgetting. This conclusion assumes, however, that our offline consciousness test identified all items that participants had consciously perceived during the earlier amnesic shadow periods. However, participants in Experiment 1 might have been able to report the masked items if we had simply asked them to do so immediately during the amnesic shadow period. To exclude this possibility, Experiment 2 adopted a maximally sensitive trial-by-trial online consciousness check to probe for awareness of the item immediately upon its presentation.

**Fig. 4:**
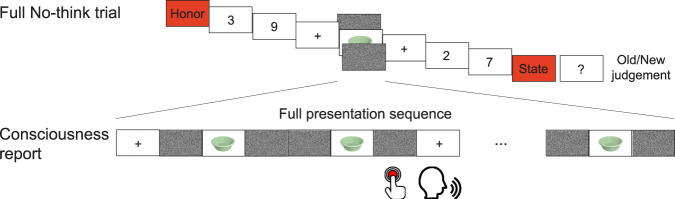
The online consciousness check procedure used in Experiment 2. We inserted the subliminal reactivation of Bystanders between two TNT trials (No-think trials in the figure). The whole series, which lasted 6 s, involved a fixed procedure which contained six repetitions of a 233 ms fixation cross, four 183.3 ms white noise masks, and two 16.7 ms cue pictures. Participants judged whether they could identify the cue object by a key press and verbally reported the content of the picture. At the end of the full trial, a question mark appeared, prompting participants to judge whether the object they been exposed to was old or new.

In this new online procedure, participants judged their consciousness state for every item. During the subliminal bystander exposures, participants pressed a button to indicate immediately whether they could consciously identify the item; if so, they verbally reported what they saw. This procedure eliminates doubt about whether a given exposure might have been perceived. In addition, after the full No-think trial had ended, a question mark prompted participants to judge whether the item they had identified was old or new (Fig. 4). To ensure that the answer to the latter episodic recognition judgments was not always “yes”, we included novel foils trials (hereinafter called “novel” trials). During these trials, instead of presenting a studied Bystander cue, we subliminally exposed an entirely novel cue object. Because the main goal of Experiment 2 was to firmly establish the subliminal nature of the amnesic shadow effect, we eliminated the supraliminal condition. To further bolster confidence that the items were truly unconscious, we adopted strict subject and item exclusion criteria based on our indices of conscious awareness.

**Fig. 5:**
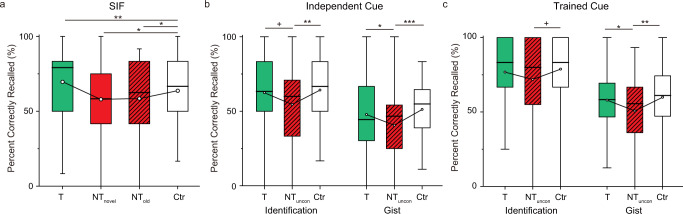
Results from Experiment 2. **a** Percentage of targets recalled for the Think/No-think (TNT) pairs (*n* = 48). Retrieval suppression consistently disrupted recall performance, causing suppression-induced forgetting in the unconscious No-think condition (T, Think; NT_novel_, No-think novel; NT_old_, No-think old; Ctr, Control). This suppression-induced forgetting effect was unaffected by whether cues to old (t(47)= −2.15, *p* = .037, Cohen’s d = 0.31) or novel (t(47)= −2.60, *p* = .013, Cohen’s d = 0.37) Bystanders were exposed during the shadow period for No-think trials. **b** Percentage of Bystander images that were recalled according to our Identification and Gist measures on the independent-cue tests. Unconscious cue exposure between two No-think trials caused forgetting of the reactivated memories linked to those cues (Identification: t(47)= −2.74, *p* = .009, Cohen’s d = 0.39; Gist: t(47)= −3.82, *p* < 0.001, Cohen’s d = 0.5). **c** Percentage of Bystander images that were recalled according to our Identification and Gist measures on the trained-probe tests. Unconscious cue exposure between two No-think trials caused forgetting of the reactivated memories linked to those cues (Identification: t(47)= −1.94, *p* = 0.059, Cohen’s d = 0.28; Gist: t(47)= −3.29, *p* = 0.002, Cohen’s d = 0.48). The boxes in box plots show the IQR and the median. Whiskers in box plots represent the minimum and maximum in the dataset. White dots represent the means for each condition. The asterisks represent significant differences (^+^*p* < 0.06; *p < 0.05, **p < 0.01, Two-tailed *t* test). Source data are provided as a Source Data file.

First, we verified that suppression-induced forgetting occurred, despite our introduction of an online consciousness-checking task. Replicating prior work, recall accuracy for TNT pairs varied significantly across the four conditions (i.e., the Think, No-think unconscious-old No-think unconscious-novel, and Control conditions) (Fig. 5a, F(3,141) = 11.97, *p* < 0.001, η_p_^2^ = 0.20, see also Supplementary Table 3 for details). Critically, retrieval suppression impaired recall performance for No-think items compared to that observed for Control items: Significant SIF arose regardless of whether intervening Bystander exposures presented previously studied cues (i.e. “old cues; No-think unconscious old vs. Control: t(47)= −2.15, *p* = 0.037, Cohen’s d = 0.31) or new foil cues (i.e. “novel” cues; No-think unconscious novel vs. Control: t(47)= −2.60, *p* = 0.013, Cohen’s d = 0.37), suggesting that suppression-induced forgetting occurred. In contrast, retrieval of items during Think trials increased recall performance (Think vs. Control: t(47)= 2.82, *p* = 0.007, Cohen’s d = 0.41).

Next, we used the findings from our trial-by-trial online consciousness check to quantify the extent to which participants were conscious of the subliminally reactivated Bystander cues. Participants failed to identify the subliminally presented item on 87.38% of the items, on this task. Thus, only 12.62% of the subliminal presentation items were reported visible and correctly named (Table 1). We further examined the episodic recognition accuracy of reported items (as measured at the end of each trial) and found high accuracy for items reported to be consciously perceived ~90% (Table 2). After eliminating the 12.62% of the items that participants identified, recognition performance for the remaining 87.38% of Bystanders was exceptionally low (43.02%) and indeed lower than chance (t(47)= −5.49, *p* < 0.001, Cohen’s d = 0.79). To the extent that the main determinant of conscious awareness is the introspective judgment that we are aware of a stimulus^20^, our findings imply that the remaining subliminally presented items were truly unconscious.

Having removed items that were consciously perceived, we next calculated the critical amnesic shadow effect on unconscious Bystanders. We performed a 2 (cue type: trained cue vs. independent cue) × 3 (suppression status: Think-unconscious, No-think-unconscious, and Control) repeated measures ANOVA separately on the Identification and Gist accuracy of Bystander target recall performance (Fig. 5b, c, see also Supplementary Table 4 for details). For the Identification accuracy measure, Bystander recall varied due to our manipulation as reflected in a main effect of suppression status (F(2,94) = 4.79, *p* = 0.010, η_p_^2^ = 0.09). Although overall recall varied across our two cue types (F(1,47) = 16.74, *p* < 0.001, η_p_^2^ = 0.26), cue-type did not interact with suppression status (F(2,94) = 0.24, *p* = 0.787, η_p_^2^ = 0.01), showing similar amnesic shadow effects under our trained and independent cues. Based on our a priori prediction of shadow-induced forgetting, we compared recall in the No-think-unconscious condition with that of the Control condition. Supporting our central hypothesis, and replicating Experiment 1, we found significant forgetting on the independent cue test: No-think Bystanders were recalled more poorly than were Control Bystanders (t(47)= −2.74, *p* = 0.009, Cohen’s d = 0.39) as also more poorly than were Think Bystanders (t(47)= −2.01, *p* = 0.051, Cohen’s d = 0.29). Although not significant, we detected a trend of shadow-induced forgetting in trained-cue retrieval (No-think vs. Control: t(47)= −1.94, *p* = 0.059, Cohen’s d = 0.28). We found no memory improvement for Think Bystanders relative to Control Bystanders (independent-cue retrieval: t(47)= −0.48, *p* = 0.637, Cohen’s d = 0.07; trained-cue retrieval: t(47)= −0.61, *p* = 0.543, Cohen’s d = 0.09).

For the Gist accuracy, the same 2 by 3 repeated measures ANOVA revealed significant main effects for suppression status (F(2,94) = 11.24, *p* < 0.001, η_p_^2^ = 0.20) and cue type (F(1,47) = 10.05, *p* = 0.003, η_p_^2^ = 0.18). In line with the findings from the Identification measure, subliminal memory reactivation within the amnesic shadow window induced by No-think trials consistently impaired the reactivated Bystanders, when compared with recall in the Control condition (independent-cue retrieval: t(47)= −3.82, *p* < 0.001, Cohen’s d = 0.55; trained-cue retrieval: t(47)= −3.29, *p* = 0.002, Cohen’s d = 0.48) and also when compared to recall for Think Bystanders (independent-cue retrieval: t(47)= −2.01, *p* = .050, Cohen’s d = 0.29; trained-cue retrieval: t(47)= −2.39, *p* = .021, Cohen’s d = 0.34). The findings of Experiment 2 thus demonstrated that both coarse and detailed information about unwanted memories could be disrupted simply by subliminally presenting reminders to them in the amnesic shadow window induced by the suppression of an independent memory.

### Analysis of the unconscious amnesic shadow effect across two experiments

**Fig. 6:**
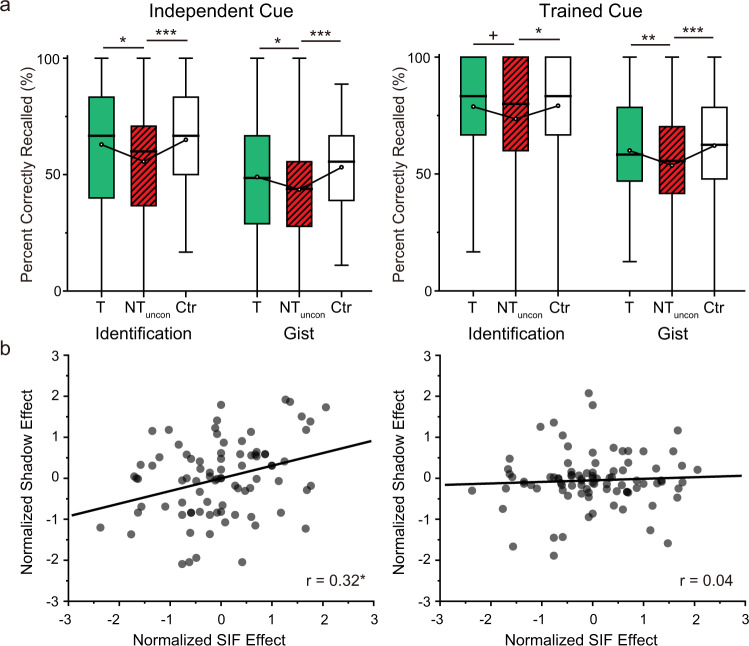
Combined analyses of Experiments 1 and 2. **a** Reduced percentages of Bystander images in the independent- (*n* = 88; Identification: t(87)= −3.74, *p* < 0.001, Cohen’s d = 0.40; Gist: t(87)= −4.44, *p* < 0.001, Cohen’s d = 0.47) and trained-cue (Identification: t(87)= −2.44, *p* = .017, Cohen’s d = 0.26; Gist: t(87)= −4.13, *p* < 0.001, Cohen’s d = 0.44) tests (T, Think; NT, No-think; Ctr, Control). The boxes in box plots show the IQR and the median. Whiskers in box plots represent the minimum and maximum in the dataset. White dots represent the means for each condition. The asterisks represent significant differences (+*p* < 0.06; *p < 0.05, **p < 0.01, Two-tailed *t* test). **b** Correlation between the suppression- and shadow-induced forgetting effects combining across the two experiments. (Left) The degree of suppression-induced forgetting on TNT pairs predicted the shadow effect in the Identification measure (r-skipped = 0.32, [0.09, 0.48] bootstrapped 95% CI). (Right) The degree of suppression-induced forgetting did not predict the shadow effect on the Gist recall measure (r-skipped = 0.04, [−0.18, 0.24] bootstrapped 95% CI). The asterisk (*) represents statistical significance at *p* < 0.05. Source data are provided as a Source Data file.

To validate the unconscious amnesic shadow effect, we removed the subjectively perceived unconscious Bystanders for each participant and combined the participants from Experiments 1 and 2. This analysis demonstrates that the amnesic shadow disrupted the identification and gist recall for unconsciously reactivated Bystander memories (Fig. 6a). The shadow-induced forgetting was most robust when memory for Bystanders was tested with the independent-cue retrieval (Identification: t(87)= −3.74, *p* < .001, Cohen’s d = 0.40; Gist: t(87)= −4.44, *p* < 0.001, Cohen’s d = 0.47), but also was significant when memory was tested with the trained-cue that was subliminally exposed during the shadow period (Identification: t(87)= −2.44, *p* = 0.017, Cohen’s d = 0.26; Gist: t(87)= −4.13, *p* < 0.001, Cohen’s d = 0.44). Overall, the amnesic shadow induced by retrieval suppression of a neutral memory is able to disrupt an independent emotional memory that was subconsciously reactivated close in time.

In Experiment 1, we observed a correlation between the suppression-induced forgetting effect for our neutral word pairs and the amnesic shadow effect for Bystanders in the conscious condition. But the same correlation was not significant in Experiment 2 (Identification: r-skipped = 0.09, [−0.26, 0.41] bootstrapped 95% CI; Gist: r-skipped = −0.07, [−0.42, 0.27] bootstrapped 95% CI). We speculated that this might arise because the amnesic shadow effect relies not only on the suppression of the hippocampal functions (induced by retrieval suppression on NT pairs) but also on how effectively our Bystanders were reactivated by their cues, an outcome that should determine its reliance on the hippocampal functions. Variability in the strength of reactivation created by Bystander cues might reduce the correlation between suppression-induced forgetting and the amnesic shadow effect. To increase the power of the correlation analysis, we combined participants from the two experiments. Indeed, with this larger sample size, on our Identification measure, suppression-induced forgetting correlated with the amnesic shadow effect on unconsciously reactivated Bystanders (Fig. 6b, r-skipped = 0.32, [0.09, 0.48] bootstrapped 95% CI). However, the same correlation was not significant when we examined the Gist measure (r-skipped = 0.04, [−0.18, 0.24] bootstrapped 95% CI). Together, these findings suggest that, as with consciously reactivated Bystanders, unconsciously reactivated memories show amnesic shadow effects that are linked to the efficacy of retrieval-suppression processes, as reflected in suppression-induced forgetting.

## Discussion

Our findings show that unpleasant memories can be forgotten by simply cuing them unconsciously during a time window in which hippocampal activity is likely to have been down-regulated. We took advantage of findings that suppressing retrieval induces anterograde and retrograde amnesia for unrelated memories encoded or reactivated near in time to suppression^6,10^. Building on this amnesic shadow finding, we reasoned that if people suppressed entirely neutral materials (e.g., simple neutral word pairs), it should induce forgetting of upsetting memories reactivated subliminally within the amnesic shadow period. Our findings support this unconscious shadow-induced forgetting prediction. Importantly, shadow-induced forgetting didn’t occur only for the reminder cues that we subliminally re-exposed; rather, the subliminally reactivated memory was also less accessible when tested with an independent cue that was not subliminally re-exposed. This cue-independent forgetting implies that the memory itself suffered generalized forgetting that arose from its reactivation during the shadow period. Thus, we induced people to forget an unpleasant memory without ever being consciously aware of the reminders that triggered the forgetting, or any intention to forget these experiences.

To ensure that the amnesic shadow effect was entirely unconscious, our two experiments employed strict controls to exclude conscious items. Experiment 1 used an offline awareness test whereas Experiment 2 adopted an online trial-by-trial awareness test, both with subjective ratings and objective recognition indices. In Experiment 1, we ensured that performance on the forced-choice recognition tests at the individual subject and group levels were at the chance level. On that basis, we excluded items that were subjectively reported as visible. To obtain an assessment of consciousness more directly tied to individual trials, Experiment 2 asked participants to indicate, on every trial, awareness immediately as they experienced it, and to report the content of the conscious item if one was experienced. This manipulation proved remarkably effective at isolating true awareness, as the reported visible items were nearly perfectly recognized on the recognition test (Table 1). Our two experiments consistently observed the shadow-induced forgetting effects in the unconscious condition, even after excluding all subjectively reported conscious items. As such, awareness of reminders appears to not be a prerequisite for shadow-induced forgetting to happen.

The ability to induce forgetting without participants’ awareness of the re-exposure process provides potentially significant clinical and theoretical advantages. These advantages flow directly from our use of the amnesic shadow phenomenon to create a window of vulnerability during which memories can be disrupted. Clinically, the most significant advantage lies with ability to induce forgetting by suppressing retrieval of an entirely unrelated, emotionally neutral memory. For example, for a traumatic automobile accident, a random set of word-word pairs (i.e., the TNT pairs) could be used for retrieval suppression while subliminally inserting a reminder picture (i.e., the Bystander cue), such as a picture of the type of car involved, between two suppression trials to reduce vivid intrusions of the accident (i.e., the Bystander target). This generalization property enables emotional memories to be modulated without perceptual awareness. Thus, our findings provide a novel method capable of bypassing the unpleasantness of consciously re-exposing people to unwelcome content, as occurs in conventional psychotherapeutic treatments for trauma-related psychiatric disorders. Theoretically, subliminal reactivation of target memories provides other advantages. Because participants were unconscious to the interpolated Bystander cues, our procedure rules out theories that might attribute the amnesic shadow to demand characteristics. One might hypothesize, however, that participants learn to withhold the recall of some bystanding memories on the final test, based on their conscious association of each item to adjacent trials and assumptions about what the experimenter wanted. This possibility is entirely excluded in our unconscious reactivation method: because participants could no longer realize the identity of each Bystander cue, it is impossible for them to adopt such a strategy. Our findings that subliminally reactivated Bystander memories are disrupted by the amnesic shadow is thus free of the influence of the experimenter effects^21^.

A critical manipulation in our study is using Bystander cues to reactivate the target memory, rather than directly presenting the aversive scenes in the amnesic shadow. The significance of this manipulation is three-fold. First, in masked cuing procedures, associative rather than single-item memory has been found to engage the hippocampus during encoding and retrieval^8,12,13^; likewise, during the unconscious state of slow-wave sleep, associative cuing, such as by contextual sounds or odors, has been found to promote consolidation of hippocampus-dependent memories^22,23^. Because hippocampal engagement is thought to be a prerequisite for the amnesic shadow to take effect^11^, associative cuing is more likely to satisfy this requirement. Second, outside the laboratory, it is never possible to present a full “memory” to participants or patients. In practice, all that we ever would have in a therapeutic setting are cues to the memory, varying in their directness and emotionality. For example, we may have a picture of a gun, or even of another soldier who has been shot, and this may be a strong cue for the soldier’s own trauma. But it is not the same thing as presenting the full “memory” to them. As such, it is vital that our study shows that presenting a relatively remote, neutral cue is sufficient to induce forgetting. Finally, selecting emotionally neutral cues to elicit the unwanted memory reduces the potential for distress to patients, should the stimulus be seen.

Despite the advantages discussed above, several issues must be examined before clinical application is considered. First, based on the hypothesis that the amnesic shadow is induced by inhibitory control over hippocampal activity^6,10^, the forgetting effect on unconsciously presented items may be restricted to hippocampus-dependent traces. To the extent that the stimuli involved in an unpleasant event have undergone affective conditioning, they may continue to evoke emotional responses even after shadow-induced forgetting occurs, given the reliance of affective conditioning on the amygdala. Moreover, the amnesic shadow may not affect familiarity-based recognition of the affected content, and may primarily disrupt context-based recollection of the event, the latter of which is believed to be more hippocampally dependent^6^. In addition, given that our shadow-induced forgetting effects occurred for recent memories acquired on the same day as retrieval suppression, it remains unclear whether similar effects occur for memories that have undergone synaptic or systemic consolidation^24^ prior to exposure within the amnesic shadow. Second, our masking procedure failed to block consciousness of Bystander pictures occasionally. Although the consciously perceived items can be eliminated from analyses to test our theoretical hypothesis, clinical applications demand even more thorough and effective masking procedure to ensure fully unconscious forgetting. A procedure that blocks consciousness while maximizing memory reactivation is preferred. Nevertheless, combining the current approach with other methods may exploit and magnify the strengths of each. Our unconscious forgetting procedure could be combined with conventional treatments to produce synergistic forgetting effects and to prevent premature drop out of treatment. For example, applying our procedure before exposure therapy may help relieve the distress due to excessive traumatic memory intrusions, whilst providing an opportunity to extinguish conditioned emotional responses. In addition, recent studies have used neuroimaging and machine learning methods to develop unconscious neural reinforcement interventions that impacted physiological activity to feared stimuli^25^. Our procedure, could provide a strong complement to such a procedure, focused on the mitigation of intrusive episodic memories.

Whereas its effects are meant to be unconscious to participants, our subliminal reactivation procedure aims to activate the target memory rather than to prevent memory reactivations. This, in fact, is a critical prerequisite for the amnesic shadow to work. It has been hypothesized that the amnesic shadow arises because retrieval suppression temporarily disrupts hippocampal function, rendering traces reactivated in this window vulnerable. Any changes to the subliminally exposed memory should only happen if the cued memory recruits hippocampal functions. The predicted dependency of the amnesic shadow on hippocampal processing of Bystander items motivated Hulbert et al.’s use of episodic encoding^6^, our prior use of episodic retrieval^10^, and our current use of associative memory reactivation to induce forgetting. Notably, achieving robust mnemonic reactivation subliminally is challenging, because the degree of reactivation depends on a complex interaction between the cue stimulus and the masks. Variability in the success of subliminal reactivation might partly obscure the association between the SIF effect and the subliminal amnesic shadow effect. However, across our two experiments, we showed a significant correlation between the two effects. We hypothesize that both suppression-induced forgetting and the amnesic shadow rely on GABAergic interneuron networks local to the hippocampus, based on relationship between hippocampal GABA concentrations and the down-regulation of hippocampal activity during retrieval suppression^26^. Sustained engagement of hippocampal inhibitory processes by the prefrontal cortex may disrupt the stabilization of the recently reactivated memory^27^. This possibility is consistent with the amnesic shadow effect occurring both anterogradely and retrogradely. However, direct evidence concerning the role of hippocampal GABA as a mechanism of the amnesic shadow effect is still lacking.

In conclusion, our studies provide strong evidence that forgetting of affective memories can be achieved completely unconsciously and without direct experiencing of aversive content, simply by inserting reminders to them into the amnesic shadow induced by suppressing entirely independent and benign memories. Moreover, our subliminal reactivation procedure ensures the memory-altering features of our procedure can be tested in a double-blind manner, wherein neither the experimenter nor the participants need be aware of which memories should be disrupted. This method holds the potential to complement existing therapeutic interventions for treating trauma and reducing high dropout rates triggered by distressing intrusions. More broadly, our findings provide evidence for the view that retrieval suppression engages a mechanism that globally suppresses hippocampal encoding and stabilization processes, disrupting even those activated hippocampal traces that do not fully enter awareness.

## Methods

The current study complies with ethical regulations for research on human participants. All the experimental procedures were approved by the Human Subject Review Committee of the Shaanxi Normal University. The experimental procedures have been preregistered (https://osf.io/5c2hf for Experiment 1 and https://osf.io/m3bhv for Experiment 2) and were performed with no deviations to the pre-registration.

### Participants

The sample size in Experiment 1 was determined in advance via a power analysis on the amnesic shadow effect in our previous study using a similar procedure. The power analysis yielded a sample size of 40 adults. Participants (aged 19–22, 32 females) were healthy Chinese adults who were required to have normal or corrected-to-normal vision. Two additional participants were excluded due to above-chance level recognition performance in the objective consciousness check as described in the Procedure section. The sample size in Experiment 2 was determined in advance via power analysis on the overall amnesic shadow effect in Experiment 1 (power = 85%, α = 0.05). We recruited a sample of 48 healthy adults (aged 18–29, 39 females) with normal or corrected-to-normal vision. No participants were excluded based on the results of the consciousness check test. Participants received monetary compensation for their time ($5 per hour) and provided informed consent before participating.

### Materials

The stimuli included a set of verbal word-pairs to implement the TNT task, and two sets of word/picture-picture pairs to be used as Bystander stimuli. We constructed 48 critical TNT paired associates. Each TNT pair was composed of two 2-character Chinese words (e.g., “legend – reason”). Each of these words was neutral in valence, as established by Xu et al.^28^ and independent subject rating. The cue and the target for each pair were semantically unassociated with each other, as established by agreement between the three experimenters. We constructed two sets of 24 pairs to test for amnesic shadow effects. Each Bystander item was composed of a target picture and two cues with which it was paired (in the form of A-X and B-X, where X was the target picture). Specifically, 24 Bystander target pictures depicting affective scenes were selected from Küpper and colleagues^29^. These pictures, originally taken from the International Affective Picture System and online sources, included themes such as physical and sexual assault, witnessing injuries and death, natural disasters, and serious accidents^10^. One set of Bystander cues used 24 object pictures. To simulate natural situations associated with involuntary trauma recall, each object cue resembled an item embedded in its paired scene. The other set of Bystander cues used 24 neutrally-valenced 2-character Chinese words^28^. These word cues were not related to the scene, as determined by the judgment of the three experimenters.

The two sets of Bystander cue-target pairs were studied and trained in separate lists. In the Think/No-think (TNT) phase, the object cues appeared embedded between TNT trials and serve as the Trained cues, whereas the word cues did not appear during the TNT phase, serving instead as Independent cues. Items from the TNT pairs were semantically unassociated with cues or targets from the Bystander pairs. The TNT and Bystander pairs were each divided into four subsets (TNT: 12 items per condition; Bystander: 6 items per group). Each subset of TNT pairs was yoked to a fixed subset of Bystander pairs (e.g., when subset one was in the NT condition, the yoked Bystanders were in the NT condition). The assignment of TNT item sets (along with their bystander) to experimental conditions was counterbalanced across participants.

In Experiment 1, the TNT and Bystander pairs were used in one of four conditions: Think unconscious, No-think conscious, No-think unconscious, and Control. In Experiment 2, we used three subsets of critical Bystander items throughout the three experimental phases. We selected another 12 object pictures to use for the offline consciousness check task. Implementing the online consciousness check task in Experiment 2 also required a further set of foils composed of object cues from unstudied Bystander pairs. Thus, in total, for each participant in Experiment 2, across the Bystander exposure task, the Bystander final test, and the Bystander consciousness check tasks, we used 18 double-cue/one-target Bystander items, and 6 Bystander object cues. Stimulus presentation and experimental manipulation were carried out using Matlab 2019b with the PsychToolbox-3 extensions^30^.

### Procedure

#### Experiment 1

Participants studied three sets of cue-target pairs, including two Bystander pair sets (where set one had the form A-X, and set two, B-X) and one TNT pair set. To ensure comparative memory strength for pairs within the same set and to avoid memory integration of pairs across different sets, the three sets were studied separately in a fixed order: the trained-cue Bystander pair set first (the object-scene pairs), the independent-cue Bystander set second (the word-scene pairs), and the TNT pairs last (the word-word pairs).

First, the trained-cue Bystander pair set (i.e., 24 object-scene pairs) was studied. Twenty-four object-scene pairs were presented to participants one at a time, each for 3 s (interstimulus interval = 1 s). Test-feedback cycles followed, in which each cue appeared alone for up to 5 s and participants judged whether they could retrieve the corresponding scene or not by pressing one of two keys. When a key was pressed or when the response window expired, the target scene appeared to the right side of the cue. Participants then reported whether they had retrieved the target picture correctly by pressing one of two keys within 5 s. Pairs that were self-reported as correctly recollected were eliminated from the subsequent test-feedback cycles. Test-feedback cycles continued until all pairs were correctly recollected. Next, the independent-cue Bystander set (i.e., 24 word-scene pairs) were studied, using the same procedure. To avoid integration of the two Bystander sets, participants were informed that the target pictures would be the same as those in the first set. They were instructed to study the new set without thinking of the first set and to avoid thinking of the three items (i.e., two cues and one common target) together. Finally, the 48 TNT word-word pairs were studied, using the same procedure as for the Bystander pairs.

Two critical manipulations—the Think/No-think task and conscious/unconscious memory reactivation—were interleaved in this phase. The Think/No-think manipulation was performed on TNT word pairs. Our aim in using the TNT task was to induce the amnesic shadow using “No-think” trials (discussed shortly). Conscious or unconscious memory reactivation was performed by presenting Bystander cues within the amnesic shadow intervals during the TNT task. Participants were notified about the subliminal presentation of Bystander cues beforehand.

Thirty-six (12 pairs from each of three subsets) from the 48 word-pairs that we trained during the learning phase were involved in this phase. Each trial in this phase presented a single cue from one of the pairs for 4 s, which participants were instructed to view continuously. Cues from one of the subsets were presented in green (Think trials) and cues from the other two subsets were presented in red (No-think-conscious and No-think-unconscious trials). For Think trials, participants were instructed to recall the associated target word upon cue onset and to think of it silently for the full 4 s. For No-think (conscious and unconscious) trials, participants were asked to avoid thinking about the associated target word while sustaining their attention on the cue word for the full 4-s duration. Procedurally No-think-conscious and No-think-unconscious trials did not differ during the No-think task itself, but only differed by virtue of the Bystander task done in its vicinity (to be discussed next). For the No-think task, the standard direct suppression instructions were used^31,32^. These instructions emphasized that participants should try to stop retrieval of the target word while avoiding replacing the target with any other diversionary thoughts or images^33^. Cues from the fourth subset of trained pairs did not appear during the TNT phase. These pairs, which were learned at the same time as the Think and No-think pairs served as a Control condition for the TNT manipulation, enabling us to estimate what final memory performance would be, given that neither retrieval nor suppression had been performed on pairs.

During the TNT task, we reactivated the target memories of three subsets of Bystander pairs by presenting their retrieval cues in between Think trials or between No-think trials (to induce double-side shadowing, which has been found to be larger than single-side shadowing^6^). Notably, only object cues were presented as Bystanders (hereinafter referred to as Trained cues). No word cues for the relevant scenes appeared during the TNT phase; these word cues were independent of the TNT manipulation (hereinafter referred to as Independent cues). The cues from the three Bystander subsets underwent different manipulations. To examine the amnesic shadow effect, cues from one Bystander subset were each presented between two Think trials and cues from the other two Bystander subsets were each presented between two No-think trials (No-think trials have been found to induce an amnesic shadow that is disruptive to memories reactivated close in time to them). To test the influence of consciousness on the amnesic shadow effect, the object cues from Think Bystanders and one subset of No-think bystanders were presented subliminally. We used the subliminal memory reactivation procedure from Degonda et al.^8^. Specifically, the object cue (S) was presented 12 times within 6 s for 16.7 ms (the total presentation duration was thus 2 s). Each cue was forwardly and backwardly masked by a 183.3-ms white Gaussian noise mask (M). A 233-ms fixation cross (F) was presented 6 times within the 6 s separating every two series of M-S-M sequences. Overall, one trial contained six continuous repetitions of the stimulation sequence of F-M-S-M-M-S-M. The fixation cross (F) would occasionally change into a vertical/horizontal bar, and participants’ task was to report its occurrence by pressing a key. Each of the items in the remaining subset of No-think Bystanders, which was used as a conscious comparison, was presented uninterrupted on the screen for 2 s, during which participants were encouraged to recognize this cue object without reporting. Notably, when Bystander cues appeared, participants were not instructed to retrieve or suppress the target scene picture in any condition. The fourth subset of the Bystander pairs did not appear in the TNT phase; these pairs, which were learned at the same time in the learning phase, served as control condition that allowed us to estimate, on the later test, retention of pairs that had never been reactivated during the TNT phase.

Before and after each Bystander, we inserted “buffer” intervals during which a series of 2-3 digits were presented on the screen. Participants classified each digit according to whether it was odd or even by pressing one of two keys. Each digit stayed on the screen for 0.9 s and was interleaved by a blank screen for 0.15 s. This procedure ensured that the same task was performed before and after every Bystander cue in all conditions; thus, Bystanders embedded between two No-think or two Think trials were nonetheless matched with regard to any task-set switching requirements before or after the Bystander exposure^6,10^, holding constant any interference such task transitions may cause. Taken together, the stimulation sequence for a full trial was “TNT task – buffer task – Bystander task – buffer task – TNT task”, followed by a 2-s fixation cross. Trials from different conditions appeared in a random order. Each TNT and Bystander task repeated eight times in eight blocks. Because one Bystander cue was embedded between two TNT cues, each Bystander pair was paired with a fixed group of two TNT pairs in all repetitions.

Following the TNT phase, participants received two tests in a fixed order. A cued-recall test was used as in previous studies^10,29^, which presented the cues for participants to verbally report the content of the target scene within 15 s (interstimulus interval = 1 s). First, we tested participants’ memories for the Bystander scenes, once using the object cue (trained cue) and once using the independent (word) cue, in separate blocks. We tested half of the participants with the trained cue first and the other half with the independent cue first. Next, we tested participants’ memory for the TNT pairs. Each cue word appeared on the screen, one at a time, and participants wrote down its associated target. The test of TNT pairs was self-paced.

At the end, we checked the participants’ consciousness level of the subliminally presented Bystander cues. The 6 Think unconscious and the 6 No-think unconscious Bystanders were included along with 12 novel object pictures. To reproduce the unconscious Bystander task, we used the same stimulation sequence of 6 repetitions of F-M-S-M-M-S-M and participants performed the same bar discrimination task as in the main experiment. After the sequence ended, participants reported whether they could see the content of the object (S) that had appeared within the series and then made an old/new response on the object. Response time was not limited.

#### Experiment 2

Experiment 2 used the same three-phase procedure as Experiment 1. One key procedural change, however, was that in the TNT phase, participants reported their ongoing consciousness state on a trial-by-trial basis during the subliminal presentation period (Fig. 4). Specifically, during the subliminal presentation period, the Bystander object cue appeared in a sequence of 6 repetitions of F-M-S-M-M-S-M as in Experiment 1. To implement the online consciousness checking task, during this 6-s period, participants made an immediate key press whenever they could consciously perceive the cue object. Whenever participants pressed a key in this manner, they then verbally reported the content of any perceived image. Finally, at the end of each full trial, participants further made an old/new judgement on the preceding masked Bystander cue, regardless of whether the stimulus was reported to be consciously perceived. This old/new judgement was self-paced.

Implementing this new online consciousness-checking task required that we introduce a new condition to the experiment. Specifically, to accommodate the need to make an old/new episodic discrimination at the end of each trial, we needed unstudied foil objects, for this discrimination to make sense. To achieve this, we added a new set of No-think Bystander trials during which an entirely novel never-before-studied Bystander object cue was embedded between two No-think trials (hereinafter, the No-think-unconscious-novel condition). For this purpose, we designed a fourth subset of 6 Bystander pairs that participants didn’t study. We used the cue objects from these pairs as the Bystander object during No-think-Unconscious-novel trials. Rather than increasing the number of trials during the TNT phase, we simply eliminated the No-think-Conscious trials of Experiment 1 (which was not needed in Experiment 2) and replaced them with No-think-Unconscious novel trials. Because we added No-think-unconscious novel condition, for clarity we refer to the No-think-unconscious condition of Experiment 1 as No-think-unconscious-old condition, to distinguish them from No-think-unconscious-novel trials.

The testing phase adopted the same procedure as in Experiment 1. No offline consciousness check was given at the end of the experiment.

### Data analysis

We performed the consciousness check analysis for Experiment 1 in two steps. First, we calculated the old/new recognition accuracy for the subliminally presented Bystander items and foils for each participant. We then compared each participant’s performance in the forced-choice test to the one-tailed 5% cutoff (66.7%) of the chance distribution of correct choices^8^. Two participants exceeded this cutoff and were replaced. Second, for each of the remaining participants, we excluded all subjectively reported visible items.

The analysis of the consciousness check task in Experiment 2 employed a different procedure than Experiment 1. First, because we forced participants to verbally report the content of any object cues that they claimed to see, our procedure greatly cut down on participants’ tendency to randomly guess during their detection decision. This enabled us to simply eliminate items that were subjectively reported to be visible at the very beginning of data analysis. Critically, because a trial-by-trial consciousness check procedure was used, items may appear conscious in some trials and unconscious in other trials. To avoid any influence of conscious awareness, any items that had been reported as consciously perceivable even just once were excluded from analyses. After eliminating the consciously recognized items, the recognition accuracy for all participants was calculated. No participant exceeded a recognition accuracy of 68.1% for the remaining Bystander cues and therefore we retained all participants.

For TNT pairs, the percentage of correctly recalled target items was calculated for each condition. For Bystander pairs, scoring for the verbal descriptions of the target scene images was based on the criteria used in previous studies^10,29^. We included two measurements, Identification and Gist. The Identification measure counted a description as correct if it included enough detail for an independent person to identify the scene. The Gist measure calculated the percentage of gist items recollected for each image. The gist items were defined as any element pertaining to the scene’s story that could not be changed or excluded without changing the main theme. Each image contained 2 to 4 predetermined gist items^29^. The Identification measure thus reflected whether participants could recollect the overall scene, and the gist measure reflected their ability to recall meaningful details of the scene. Data analysis was performed in jamovi 1.6.23 (https://www.jamovi.org).

### Reporting summary

Further information on research design is available in the Nature Research Reporting Summary linked to this article.

## Supplementary information

Supplementary Information

Reporting Summary

## Data Availability

The data generated in this study have been deposited in OSF and are accessible at https://osf.io/384sk/. Source data are provided with this paper.

## Source data

Source Data
