# Supplementary material for: Inducing forgetting of unwanted memories through subliminal reactivation
Source: Nat Commun. 2022 Oct 30;13:6496. doi: 10.1038/s41467-022-34091-1 (PMC9618560; doi:10.1038/s41467-022-34091-1)
Supplement: Supplementary file 1 — Supplementary Information [file 41467_2022_34091_MOESM1_ESM.pdf]

Supplementary Tables

Table 1. Suppression-induced forgetting from Experiment 1 (%)

Think	No-think conscious	No-think unconscious	Control
64.79	54.79	55.83	61.67
(25.14)	(26.95)	(24.33)	(25.09)

Table 2. Shadow-induced forgetting from Experiment 1 (%)

	Independent Cue				Trained Cue			
	Think	No-think conscious	No-think unconscious	Control	Think	No-think conscious	No-think unconscious	Control
Identification	60.42	56.67	54.58	65.83	49.73	43.33	45.39	55.49
	(23.17)	(20.60)	(21.35)	(23.56)	(21.89)	(18.31)	(17.14)	(20.63)
Gist	82.08	72.50	74.17	79.58	63.72	56.74	56.56	64.79
	(19.75)	(24.03)	(20.31)	(21.18)	(18.91)	(24.37)	(18.10)	(22.28)

Table 3. Suppression-induced forgetting from Experiment 2 (%)

Think	No-think novel	No-think old	Control
69.62	55.42	56.67	61.04
(23.97)	(24.64)	(24.55)	(21.88)

Table 4. Shadow-induced forgetting from Experiment 2 (%)

	Independent Cue			Trained Cue		
	Think	No-think unconscious	Control	Think	No-think unconscious	Control
Identification	60.50	52.83	62.08	77.08	68.96	77.08
	(25.55)	(28.35)	(19.96)	(22.04)	(31.14)	(25.79)
Gist	45.22	40.10	49.44	57.21	48.93	58.37
	(24.45)	(24.42)	(16.86)	(20.19)	(24.14)	(22.68)
