# Supplementary material for: Inducing forgetting of unwanted memories through subliminal reactivation
Source: Nat Commun. 2022 Oct 30;13:6496. doi: 10.1038/s41467-022-34091-1 (PMC9618560; doi:10.1038/s41467-022-34091-1)
Supplement: Supplementary file 2 — Reporting Summary [file 41467_2022_34091_MOESM2_ESM.pdf]

nature portfolio

Corresponding author(s): Zijian Zhu and Yingying Wang

Last updated by author(s): Aug 20, 2022

Reporting Summary

Nature Portfolio wishes to improve the reproducibility of the work that we publish. This form provides structure for consistency and transparency in reporting. For further information on Nature Portfolio policies, see our Editorial Policies and the Editorial Policy Checklist.

Statistics

For all statistical analyses, confirm that the following items are present in the figure legend, table legend, main text, or Methods section.

n/a	Confirmed	
☐	☒	The exact sample size (*n*) for each experimental group/condition, given as a discrete number and unit of measurement
☐	☒	A statement on whether measurements were taken from distinct samples or whether the same sample was measured repeatedly
☐	☒	The statistical test(s) used AND whether they are one- or two-sided<br>*Only common tests should be described solely by name; describe more complex techniques in the Methods section.*
☒	☐	A description of all covariates tested
☐	☒	A description of any assumptions or corrections, such as tests of normality and adjustment for multiple comparisons
☐	☒	A full description of the statistical parameters including central tendency (e.g. means) or other basic estimates (e.g. regression coefficient) AND variation (e.g. standard deviation) or associated estimates of uncertainty (e.g. confidence intervals)
☐	☒	For null hypothesis testing, the test statistic (e.g. *F*, *t*, *r*) with confidence intervals, effect sizes, degrees of freedom and *P* value noted<br>*Give P values as exact values whenever suitable.*
☒	☐	For Bayesian analysis, information on the choice of priors and Markov chain Monte Carlo settings
☒	☐	For hierarchical and complex designs, identification of the appropriate level for tests and full reporting of outcomes
☐	☒	Estimates of effect sizes (e.g. Cohen's *d*, Pearson's *r*), indicating how they were calculated

Our web collection on statistics for biologists contains articles on many of the points above.

Software and code

Policy information about availability of computer code

Data collection	Psychtoolbox-3 (http://psychtoolbox.org) in MATLAB 2019a (The MathWorks Inc., Natrick, MA, USA) was used to collect data in this study.
Data analysis	We used custom scripts in MATLAB 2019a (The MathWorks Inc., Natrick, MA, USA) to analyze data in this study. ANOVA and t tests were performed in jamovi 1.6.23 (https://www.jamovi.org).

For manuscripts utilizing custom algorithms or software that are central to the research but not yet described in published literature, software must be made available to editors and reviewers. We strongly encourage code deposition in a community repository (e.g. GitHub). See the Nature Portfolio guidelines for submitting code & software for further information.

Data

Policy information about availability of data

All manuscripts must include a data availability statement. This statement should provide the following information, where applicable:

- Accession codes, unique identifiers, or web links for publicly available datasets
- A description of any restrictions on data availability
- For clinical datasets or third party data, please ensure that the statement adheres to our policy

The data generated in this study have been deposited in OSF and are accessed at https://osf.io/384sk/. Source data are provided with this paper.

Human research participants

Policy information about studies involving human research participants and Sex and Gender in Research.

Reporting on sex and gender	We recruited a sample of 48 healthy Chinese adults (aged 19-22, 32 females) in Experiment 1 and a sample of 40 healthy Chinese adults (aged 18 to 29, 39 females) in Experiment 2. All participants were with normal or corrected-to-normal vision.
Population characteristics	Participants were healthy adults recruited from Shaanxi Normal University, Xi'an, China. They were randomly recruited using online advertisments and are representative of young adults in China.
Recruitment	Participants were recruited through online advertisement. They were required to have normal or corrected-to-normal vision. Participants received monetary compensation for their time ($5 per hour). No other criteria were applied to screen the participants during the recruitment. No self-selection bias or other biases that were likely to impact results were present.
Ethics oversight	The current study complies with ethical regulations for research on human participants. All the experimental procedures were approved by the Human Subject Review Committee of the Shaanxi Normal University.

Note that full information on the approval of the study protocol must also be provided in the manuscript.

Field-specific reporting

Please select the one below that is the best fit for your research. If you are not sure, read the appropriate sections before making your selection.

☐ Life sciences ☒ Behavioural & social sciences ☐ Ecological, evolutionary & environmental sciences

For a reference copy of the document with all sections, see nature.com/documents/nr-reporting-summary-flat.pdf

Behavioural & social sciences study design

All studies must disclose on these points even when the disclosure is negative.

Study description	Quantitative experimental. A within-subject design was used. Specifically, Experiment 1 contains four within-subject conditions which are Think, No-think conscious, No-think unconscious, and Control. In Experiment 2, the suppression-induced effects are compared across four within-subject conditions which are Think, No-think novel, No-think old, and Control; the shadow effects are examined in three within-subject conditions which are Think, No-think, and Control.
Research sample	The sample size in Experiment 1 was determined in advance via a power analysis on the amnesic shadow effect in our previous study using a similar procedure; the sample size in Experiment 2 was determined via a power analysis on the amnesic shadow effect in Experiment 1. The power analysis yielded a sample size of 40 (aged 19-22, 32 females) adults for Experiment 1 and 48 (aged 19-22, 32 females) for Experiment 2. Participants were healthy adults recruited from Shaanxi Normal University, Xi'an, China. They were randomly recruited using online advertisments and are representative of young adults in China.
Sampling strategy	Participants were recruited and randomly assigned to different stimulus assignments. The sample size in Experiment 1 was determined in advance via a power analysis on the amnesic shadow effect in our previous study using a similar procedure; the sample size in Experiment 2 was determined via a power analysis on the amnesic shadow effect in Experiment 1 (power = .85, alpha = .05).
Data collection	The computer was used to record participants' responses during the first two experiment phases and a recorder was used to collect their verbal responses during the final recall test. During the whole experiment, an experimenter was present with the participant and would record the participant's verbal responses during consciousness check and final recall test. The experimenter did not know about the experimental conditions as the stimuli were randomly assigned to different conditions and they only recorded participants' responses. The participants did not know the stimuli condition either. They were also naive to the research question.
Timing	Mar. 17th, 2021 to Jun. 9th, 2021
Data exclusions	Two participants were excluded before data analysis to ensure that the memory reactivation in our amnesic shadow is subconscious. Specifically, we compared each participant's performance in the forced-choice test for consciousness check to the one-tailed 5% cutoff (66.7%) of the chance distribution of correct choices (see Degonda et al., 2005). Two subjects exceeded this cutoff and were replaced. The procedure of subject exclusion during consciousness check was preregistered (https://osf.io/5c2hf) before data collection.
Non-participation	No participants dropped out during the experiment.
Randomization	Participants were not allocated to experimental groups.

Reporting for specific materials, systems and methods

We require information from authors about some types of materials, experimental systems and methods used in many studies. Here, indicate whether each material, system or method listed is relevant to your study. If you are not sure if a list item applies to your research, read the appropriate section before selecting a response.

Materials & experimental systems

n/a	Involved in the study
☒	☐ Antibodies
☒	☐ Eukaryotic cell lines
☒	☐ Palaeontology and archaeology
☒	☐ Animals and other organisms
☒	☐ Clinical data
☒	☐ Dual use research of concern

Methods

n/a	Involved in the study
☒	☐ ChIP-seq
☒	☐ Flow cytometry
☒	☐ MRI-based neuroimaging
